# Macrophage activation syndrome-like in multiple myeloma patients treated with the academic CAR-T against BCMA ARI0002h

**DOI:** 10.3389/fimmu.2025.1654096

**Published:** 2025-10-16

**Authors:** Daniel Munárriz, Luis Gerardo Rodríguez-Lobato, Lucía López-Corral, Cristina Arnaldos-Pérez, Valentín Cabañas, Nieves López-Muñoz, Aina Oliver-Caldés, Juan Carlos Ponce, Juan Luis Reguera, Ana África Martín, Núria Martínez-Cibrián, Natalia Tovar, Julio Delgado, Elena Guillen, Sara Varea, Paula Rodríguez-Otero, Álvaro Urbano-Ispizua, José María Moraleda, Joaquín Martínez-López, María-Victoria Mateos, Verónica González De la Calle, Europa Azucena González-Navarro, Carlos Fernández de Larrea

**Affiliations:** ^1^ Hospital Clínic de Barcelona, Instituto de Investigaciones Biomédicas August Pi i Sunyer (IDIBAPS), University of Barcelona, Barcelona, Spain; ^2^ University Hospital of Salamanca, Biomedical Research Institute of Salamanca Instituto de Investigación Biomédica de Salamanca, Cancer Research Center Instituto de Biología Molecular y Celular del Cáncer (IBMCC), Consejo Superior de Investigaciones Científicas (CSIC) , Salamanca, Spain; ^3^ Virgen de la Arrixaca University Clinical Hospital, Instituto Murciano de Investigación Biosanitaria-Arrixaca, University of Murcia, Murcia, Spain; ^4^ University Hospital 12 de Octubre, Complutense University, Centro Nacional de Investigaciones Oncológicas (CNIO), Madrid, Spain; ^5^ University Clinic of Navarra, Center for Applied Medical Research (CIMA), Instituto de Investigación Sanitaria de Navarra (IDISNA), Centro de Investigación Biomédica en Red (CIBERONC) CB16/12/00369, Pamplona, Spain; ^6^ Virgen del Rocío University Hospital, Institute of Biomedicine of Seville (IBIS/CSIC/Centro de Investigación Biomédica en Red (CIBERONC)), University of Seville, Seville, Spain

**Keywords:** myeloma, anti-BCMA CAR T, MAS-like syndrome, IEC-HS, immunotherapy

## Abstract

**Background:**

Chimeric antigen receptor T-cell (CAR-T) therapy targeting B-cell maturation antigen (BCMA) has revolutionized multiple myeloma treatment (MM). However, managing its immune-mediated adverse events, particularly macrophage activation syndrome-*like* (MAS-*like*), remains challenging due to underreporting.

**Methods:**

This multicentre, retrospective, analytical study evaluated MM patients treated with the anti-BCMA academic product ARI0002h. MAS-*like* was defined using the University of California San Francisco (UCSF) consensus criteria. Primary endpoints included baseline characteristics, predictive factors, and survival outcomes associated with MAS-*like*.

**Results:**

Of 80 patients, 12 (15%) met the UCSF criteria for MAS-*like*. These patients presented higher ISS scores (ISS III: 54.5% vs. 15.2%; *p* = 0.006), elevated serum monoclonal components (31.3 g/L vs. 6.8 g/L; *p* = 0.004), and a higher prevalence of extramedullary disease (41.7% vs. 16.2%; *p* = 0.057). MAS-*like* typically emerged 9 days post-infusion, with elevated ferritin, followed by LDH (median 11.5 days) and hypofibrinogenemia (median 14 days). One-third of patients met all UCSF criteria, and all exhibited hypertriglyceridemia, hypertransaminasemia, and cytopenias. Histopathological examination was positive in 63% of evaluated patients. Patients who developed MAS-*like* had poorer responses (CR: 25% vs. 68%; p = 0.008) and shorter median PFS and OS (7 months vs. 21.4 months and 18 months vs. not reached, respectively; p = 0.004). Those meeting all UCSF criteria had even inferior outcomes.

**Conclusions:**

MAS-*like* is associated with poorer responses, reduced PFS and OS, especially in patients meeting all UCSF criteria. High tumour burden, including elevated monoclonal component, high ISS and extramedullary disease, seems to contribute to MAS-*like* development.

## Introduction

The development of chimeric antigen receptor T-cell (CAR-T) therapy has revolutionized the treatment landscape for multiple myeloma (MM). Thus, CAR-T therapy is considered one of the key therapeutic options, particularly for patients with relapse/refractory multiple myeloma (R/R MM). Among the principal CAR-T therapies worldwide are idecabtagene vicleucel^1^ (ide-cel) and ciltacabtagene autoleucel (1, 2) (cilta-cel), both of which have demonstrated a favorable efficacy profile. However, the management of the complications associated with CAR-T remains a significant challenge. Notably, non-relapse mortality (NRM) is higher in multiple myeloma (8.0%) compared to other hematologic malignancies such as large B-cell lymphoma (6.1%) and indolent lymphoma (5.7%) (3). Inflammation-related complications, such as cytokine release syndrome (CRS) and Hemophagocytic Lymphohistiocytosis (HLH), account for up to 25% of NRM as reported by Sidana et al. Furthermore, the immunosuppressive treatments used to manage these complications increase the risk of infections, which represent the leading cause of death in these patients (4). Unfortunately, despite the existence of various consensus definitions of CAR-T-related HLH-*like*, the ambiguous terminology and lack of strict diagnostic criteria hinder accurate diagnosis (5–8).

Secondary HLH related to cellular therapy has gained increasing recognition in recent years with the expanding use of CAR-T. Initially identified through the classical clinical features of HLH, more recently proposed diagnostic frameworks have introduced related definitions, such as Immune Effector Cell-Associated Hemophagocytic Lymphohistiocytosis-Like Syndrome (IEC-HS) and Macrophage Activation Syndrome-like (MAS-*like*). Although awareness of this manifestation is increasing, it remains insufficiently characterized in clinical trials of anti-BCMA CAR-T therapy (1, 9). Nevertheless, MAS-*like* constitutes a relatively frequent complication of BCMA-directed CAR-T therapy, with prior studies reporting an incidence of approximately 20% (7, 10). Despite growing concern, the pathogenesis of MAS-*like* remains unclear. It is characterized by a distinct cytokine profile compared to CRS, with marked elevations of interferon-γ, granzyme B, interleukin-1 receptor antagonist (IL-1RA), and interleukin-10 (11). Some patients exhibit a biphasic expansion of CAR-T cells. The first peak is predominantly composed of PD1+ T effector cells, while the second peak is characterized by T-cell factor 1 positive (TCF1+) T effector cells, which are primarily involved in memory cell responses. This second expansion phase of CAR-T cells has been associated with the development of an uncontrolled inflammatory response (12).

Controlling the inflammatory response is a key challenge in CAR-T therapy for MM. Academic approaches to CAR-T cell therapy are offering novel insights aimed at enhancing safety, minimizing neurological toxicities, and enabling point-of-care treatments where the *bench-clinic-bench* time is reduced. For example, cesnicabtagen autoleucel (13) (ARI0002h), an academic anti-BCMA CAR-T developed by Hospital Clínic of Barcelona, is administered in three different fractions (10%, 30%, and 60% of total cells), on non-consecutive days. This methodology has been employed since the development of the anti-CD19 CAR T-cell product ARI0001, with the aim of reducing systemic complications. In this regard, a low incidence of high-grade adverse events and only mild and transient neurological toxicity has been observed in ARI0002h, with no late neurotoxicity (13, 14).

With these results from the multicentre clinical trial CARTBCMA-HCB-01, the approval for use under hospital exemption was granted in Spain (13–15). Our group aimed to investigate the incidence, clinical manifestations, predictive factors and prognosis of MAS-like in patients treated with ARI0002h.

## Methods

### Cesni-cel (ARI0002h)

ARI0002h is a second-generation CD8α-TMD BCMA-4-1BBζ CAR-T product, lentivirally transduced into autologous T cells collected via peripheral blood leukapheresis (16). This CAR-T cell therapy is an academic product developed by Hospital Clínic of Barcelona in collaboration with the University of Barcelona and IDIBAPS. The CAR-T cell production was conducted at two Spanish centers (Hospital Clínic Barcelona and Clínica Universitaria de Navarra), and the infusions were performed at six different Spanish academic institutions.

### Study design and population

For this study, patients who received ARI0002h between July 2020 and January 2024 were analysed, included in clinical trial CARTBCMA-HCB-01 or under compassionate use in our institution. Inclusion and exclusion criteria of CARTBCMA-HCB-01 are detailed in the **Supplementary Material** (annex 1). Patients received intravenously an initial fractionated infusion of 3 × 10^6^ CAR T cells per kg bodyweight in three aliquots (10%, 30% and 60%, separated by 48 hours). If adverse events occurred between administrations, the remaining doses were withheld, except in cases of low-grade toxicity, in which the infusion continued after resolution. A non-fractionated booster dose of up to 3 × 10^6^ CAR T cells per kg bodyweight was administered, at least 100 days after the first infusion (13). This fractioning protocol was also employed in the clinical trial CART19-BE-01 (17) with ARI-0001, and has demonstrated limited immune-related side-effects such as CRS and immune effector cell-associated neurotoxicity (ICANS), without reducing efficacy (14).

The data collected for this study was updated until October 2024. The study was approved by the Institutional Review Board of Hospital Clínic of Barcelona and adhered to the principles of the Declaration of Helsinki.

### Definitions and outcomes of interest

MAS-*like* was defined according to University of California San Francisco (UCSF) consensus criteria: [1] ferritin rise ≥100 mg/L/h within a 24-hour period and [2] minimum fibrinogen <150 mg/dL or maximum lactate dehydrogenase >2 times the upper limit of normal or histopathological diagnosis (hemophagocytosis) (7). Patients were retrospectively assessed and catalogued according to the UCSF criteria. Definition of disease evaluation, such as overall response rate (ORR), complete response (CR), very good partial response (VGPR) and partial response (PR) was done according to International Myeloma Working Group (IMWG) (18).

The main endpoints included an analysis of baseline characteristics, PFS and OS based on patients who developed MAS-*like*. We also explored how to identify risk factors for developing MAS-*like* and to characterize the clinical manifestations of this syndrome. In survival analyses, PFS was defined from the CAR-T infusion time to disease progression or death. OS was defined as the time from the day of CAR-T administration to death from any cause.

### Variants related to HLH by exome sequencing

We performed targeted exome sequencing focusing on a panel of HLH-related genes believed to influence the predisposition to macrophage activation syndrome (MAS). The genes included in this panel are listed in **Supplementary Table 1**. Genomic DNA was extracted from peripheral blood, and library preparation was carried out according to the Agilent SureSelect protocol, with sequencing performed using the NovaSeqX platform.

Exome data were analyzed using the Galaxy platform, and quality control was performed using the FastQC tool. Reads were mapped to the reference human genome (hg38) using the BWA-MEM2 tool. Variant calling was performed with the FreeBayes tool, and the identified variants were annotated using the online Ensembl Variant Effect Predictor (19).

Variants were filtered based on their location (exonic regions plus 3 base pairs into the intronic regions), minor allele frequency (<0.04 in GnomAD, to avoid missing polymorphisms considered as risk factors) (20) and predicted impact on protein function. Variants with a frequency >1% in the general population in GnomAD that had not been reported as risk factors were excluded.

### Statistical analysis

Continuous variables were assessed using Student’s t-test or appropriate non-parametric methods. Categorical variables were summarized as frequencies and percentages, and statistical comparisons made using Fisher’s test. Median follow-up was calculated using the reverse Kaplan–Meier method. The optimal cutoff points of quantitative variables were determined using receiver operating characteristic (ROC) curve analysis.

Univariate and multivariate logistic regression models were employed to identify factors associated with MAS-*like* development. The starting point for time-to-event analysis was the CAR T-cell infusion. The development of MAS-*like* was analysed as a time-varying covariate. Cox proportional hazards regression with backward stepwise selection was utilized to identify factors associated with PFS and OS. A Cox proportional hazards model was fitted to examine the effect of MAS-*like* on patient survival. The proportionality assumption was evaluated using the Schoenfeld test, which revealed no evidence of violation of this assumption (*p* = 0.56).

All statistical tests were two-tailed, with significance set at an alpha level of 0.05. For subgroup analyses, p-values were also adjusted using the False Discovery Rate (FDR) method, in the context of a small sample size. Analyses were conducted using RStudio 2024.04.4, and GraphPad Prism 10.

## Results

### Patient characteristics

A total of 80 patients who received ARI0002h between July 2020 and January 2024 were analysed with a median follow-up of 16.5 months (IQR 9.3-21.8). Among then, 60 patients were included in clinical trial CARTBCMA-HCB-01 and 20 were treated under compassionate use, with similar inclusion and exclusion criteria. The CAR-T cell infusions were performed at six different Spanish academic institutions. The baseline characteristics of the cohort are summarized in **Table 1**. The median age of patients was 59 years, and the most frequent heavy chain isotype was IgG (51%). Extramedullary disease was observed in 20% of cases, and 38% of patients exhibited high-risk cytogenetics. The median number of prior lines of therapy was 3, with 58% of patients being triple-refractory. Additionally, 21% of patients presented with an International Staging System (ISS) stage III at the time of enrolment. After CAR-T cell therapy, ORR was 96% and CR 62% in the whole series, with a median PFS of 20 months (95% CI 13.8-23.6) and median OS not reached (95% CI 21.5-NR). CRS occurred in 81% of patients (4.6% grade ≥3), with two cases of ICANS (2.5%) grade 1 (**Table 2**).

**Table 1:** Baseline characteristics of the patients analyzed.

Variables	Total (N = 80)	MAS-*like* ^1^ (N = 12)	No MAS-*like* (N = 68)	*p*
Age, median (range)(years) Age ≥ 65, total (%)	59 (36–74) 26/80 (32.5)	55 (43–73) 5/12 (41.7)	59 (36–74) 21/68 (30.1)	0.61
Sex Female, total (%)	35/80 (44.8)	4/12 (33.3)	31/68 (45.6)	0.54
ECOG 0-1	75 (93.8)	12 (100)	63 (92.6)	0.35
ISS at inclusion, total (%) ^2^ I-II III	61/77 (79.2) 16/77 (20.8)	5/11 (45.5) 6/11 (54.5)	56/66 (84.8) 10/66 (15.2)	**<0.01**
Isotype heavy chain, total (%) IgG IgA IgM Bence Jones	41/80 (51.3) 20/80 (25) 2/80 (0.3) 17/80 (21.3)	9/12 (75) 1/12 (8.3) 0/12 (0) 2/12 (16.7)	32/68 (47.1) 19/68 (27.9) 2/68 (2.9) 15/68 (22.1)	0.12
Isotype light chain, total (%) Kappa Lambda	44/80 (55) 36/80 (45)	7/12 (58.3) 5/12 (41.7)	37/68 (54.4) 31/68 (45.6)	1
Plasma cells in bone marrow (%), median (range)	3.5 (0–100)	3 (0–100)	5.5 (0–100)	0.95
Monoclonal component serum, median (range) (g/L) Monoclonal component serum >30 g/L, total (%)	10.1 (0–90) 11/77 (14.3)	31.3 (0–90) 7/11 (63.6)	6.8 (0-71.1) 4/68 (5.8)	**<0.01** **<0.01**
Monoclonal component urine, median (range) (g/24h)	1.1 (0–100)	0.3 (0–74)	1.12 (0–100)	0.75
Involved light chains serum, median (range) (mg/L)	318 (5–7326)	472.9 (61–3373)	268.7 (5–7326)	0.48
High risk cytogenetics, total (%) del(17p)/TP53 gain(1q) t(4,14)	27/72 (37.5) 15/71 (21.1) 19/61 (31.1) 11/71 (15.5)	3/11 (27.3) 0/11 (0) 3/11 (27.3) 1/11 (9.1)	24/61 (39.3) 15/60 (25) 16/50 (32) 10/60 (16.7)	0.67 0.11 1 0.85
Plasmocytomas, total (%) Extramedullary disease, total (%)	39/80 (48.8) 16/80 (20)	9/12 (75) 5/12 (41.7)	30/68 (44.1) 11/68 (16.2)	0.06 0.06
Number of prior lines, median (range)	3 (2–10)	4 (2–9)	3 (2–10)	0.24
Triple refractory, total (%)	45/78 (57.7)	7/10 (70)	38/68 (55.9)	0.51
HSCT, total (%)^3^ Auto-HSCT, total (%)^4^	73/80 (91.3) 67/80 (83.8)	9/12 (75) 8/12 (66.7)	64/68 (94.1) 59/68 (86.8)	0.07 0.1
Bridging treatment, total (%)	44/80 (55)	7/12 (58.3)	37/68 (54.4)	1
Second dose (*booster*), total (%)	61/67 (91)	6/8 (75)	55/59 (93.2)	0.15

^1^MAS-*like*: Macrophage activation syndrome (according to consensus criteria: increase in ferritin ≥100 μg/L/h over 24 hours + either fibrinogen <150 mg/dL or LDH >2 times the upper limit of normal) or HLH, hemophagocytic lymphohistiocytosis. ^2^ISS, International Staging System. ^3^HSCT, hematopoietic stem cell transplantation. ^4^Auto-HCT, autologous hematopoietic progenitor transplantation. The bold values highlight statistically significant results.

**Table 2:** Response grade and toxicities (cytokine release syndrome [CRS] and immune effector cell-associated neurotoxicity syndrome [ICANS]) following infusion.

Variables (N = 80)	Total (N = 80)	MAS-*like* ^1^ (N = 12)	No MAS-*like* (N = 68)	*p*
ORR, total (%)	77/80 (96.3)	11/12 (91.7)	66/68 (97.1)	0.37
CR, total (%)	49/80 (61.3)	3/12 (25)	46/68 (67.6)	**0.04**
Time to best response Time in days, mean (range)	99 (20–595)	89 (22–183)	109 (20–595)	0.58
CRS^6^, total (%)	65/80 (81.3)	12/12 (100)	53/68 (77.9)	0.11
CRS grade 1 ≥2	43/65 (66.2) 22/65 (33.8)	9/12 (75) 3/12 (25)	34/53 (64.2) 19/53 (35.8)	0.86
ICANS^7^, total (%)	2/80 (2.5)	0/12 (0)	2/68 (2.9)	1
ICANS grade 1 ≥2	2/2 (100) 0/2 (0)	0/2 (0) 0/2 (0)	2/2 (100) 0/2 (0)	1

ORR, overall response rate; sCR, stringent complete response; CR, complete response; VGPR, very good partial response; PR, partial response; CRS, cytokine release syndrome; ICANS, immune effector cell associated-neurotoxicity syndrome. The bold values highlight statistically significant results.

### MAS-*like* syndrome characteristics

Among the 80 patients, 12 met the criteria for MAS-*like* (15%). These patients had a higher International Staging System (ISS) score at enrolment (ISS III 54.5 vs 15.2%, *p* = 0.006) and elevated serum monoclonal component levels compared to the controls (31.3 vs 6.8 g/L, *p* = 0.004). No other significant differences were observed, although there was a higher prevalence of extramedullary disease (41.7% vs. 16.2%, *p* = 0.057). The incidence of infection prior to infusion (<2 months) was explored and was not found to be increased. Referring to immune kinetics, CAR-T cell expansion and soluble BCMA levels in peripheral blood were analyzed, without revealing any statistically significant differences between patients who developed MAS-like and those who did not.

The characteristics of MAS-*like* are summarized in **Table 3**. The syndrome predominantly occurred following the administration of the third fraction of the first dose (83.3%) and approximately 9 days after the first aliquot, characterized by an initial increase in ferritin, which reached its peak at a median of 5 days. This increase was followed by a rise in lactate dehydrogenase (LDH) levels (median 11.5 days), and subsequently by hypofibrinogenemia (median 14 days). The moment when at least two criteria were met was approximately with a median of 10 days (IQR 9.5-14.5). The LDH criterion was met more frequently than the fibrinogen criterion (83.3% vs. 60%). One-third of the patients met all three criteria during the MAS-*like* episode (33%). In this cohort, the maximum ferritin level (median 10991 ng/mL, IQR 6357-17334) coincided with the minimum fibrinogen level (median 0.81 g/L, IQR 0.77-2), showing a weak negative correlation (-0.38, *p* = 0.401) (**Supplementary Figure 1**).

**Table 3:** Clinical and analytical characteristics of patients affected by MAS-*like*.

Variables	MAS-*like* (N = 12)
Symptomatic, total (%)^1^	11/12 (91.2)
Number of aliquots at event 2 3	2/12 (16.7) 10/12 (83.3)
Ferritin criterion, total (%)	11/12 (91.2)
Time to ferritin criterion post-infusion (days), median (range)	9 (3–20)
Ferritin at the onset of increase (ng/ml), median (range)	892.6 (385–5675)
Maximum ferritin (ng/ml), median (range)	10991 (3304–82300)
Time to maximum ferritin post-infusion (days), median (range)	14 (8–21)
Biphasic ferritin elevation, total (%)	1/12 (8.3)
LDH criterion, total (%)^2^	10/12 (83.3)
Time to LDH criterion post-infusion (days), median (range)	11.5 (7–20)
Fibrinogen criterion, total (%)	6/10 (60)
Time to fibrinogen criterion post-infusion (days), median (range)	14 (10–18)
Minimum fibrinogen (g/l), median (range)	0.81 (0.6-0.82)
Hypertriglyceridemia, total (%)^3^	9/9 (100)
Maximum triglycerides (mg/dl)	439.5 (237–769)
Hypertransaminasemia, total (%)^4^	12/12 (100)
Maximum AST (U/l)^5^	145.9 (63–9774)
Number of cytopenias, total (%) 2 3	5/12 (41.7) 7/12 (58.3)
Anatomopathological diagnosis, total (%)	5/8 (62.5)

^1^Symptomatic: presence of fever >38°C/hepatomegaly/splenomegaly, ^2^LDH: lactate dehydrogenase, ^3^AST: aspartate aminotransferase.

All patients exhibited classical MAS features, including hypertriglyceridemia (>150 mg/dL), hypertransaminasemia (AST >30 U/L), and ≥ 2 cytopenias (defined as haemoglobin <10 g/dl, platelets <100×10^9/l and leucocytes <4×10^9/l). Pancytopenia was found in 58.3% of patients (7 out of 12). Histopathological examination, based on bone marrow aspiration or tissue biopsy, was positive in 5 out of 8 patients evaluated (62.5%).

### Association with HLH DNA variants

Sequencing of exonic regions of an HLH-related gene panel was performed on 44 patients (12 who developed MAS-*like* and 32 who did not). We identified 7 genetic variants in patients who developed MAS-*like* and 23 in the control group (**Supplementary Table 2**). Key variants were observed in the *PRF1* and *UNC13D* genes. For instance, the *PRF1* Ala91Val variant, known to impair perforin activity (20), was found in 16.7% of patients with MAS-*like* and 12.5% of patients without MAS-*like*. Statistical analysis did not show a significant difference, suggesting that other factors might contribute to MAS-*like* development alongside genetic predispositions.

### MAS-*like* treatment

Treatment was primarily based on tocilizumab (an anti-IL-6 agent), corticosteroids and anakinra (an anti-IL-1 agent). Most patients (75%) received at least one dose of tocilizumab at the standard dose of 8 mg/kg, with 37.5% and 12.5% receiving two and three doses, respectively. Dexamethasone was administered in half of the cases, with daily doses ranging between 10 and 20 mg and a median duration of 3 days. Nearly half of the patients (42%) received combination therapy. Four patients (33%) required anakinra at 8 mg/kg/day, with a median duration of 5 days. No additional drugs commonly employed in MAS-like were used.

### Factors associated with the development of MAS-*like*

In the univariate analysis (**Supplementary Table 3A**), an ISS score of 3 at screening was associated with an increased risk of developing MAS-like (OR: 6.72, 95% CI: 1.72–26.3, p = 0.006), as was a higher serum monoclonal component (OR: 1.06, 95% CI: 1.02–1.1, p = 0.003). The predictive performance of the serum monoclonal component for MAS-like syndrome was further assessed using ROC analysis, yielding a T value of 4.18 (p = 0.00007). A threshold of 29.4 g/L demonstrated the highest predictive accuracy (AUC: 0.7645, 95% CI: 0.5831–0.9458). Additionally, serum monoclonal component levels greater than 30 g/L were associated with a significantly increased risk (OR: 14.6, 95% CI: 3.28–65.4, p = 0.0004). The presence of extramedullary disease was associated with a non-significantly increased risk (OR: 3.7, 95% CI: 0.99–13.80, p = 0.051). In the multivariate analysis (**Supplementary Table 3B**), serum monoclonal component greater than 30 g/L remained statistically significant (OR: 10.1, 95% CI: 1.67–61.5, p = 0.01).

### Impact of MAS-*like* development on patient clinical outcomes

The ORR of the cohort was 96%, with 89% of patients achieving at least a VGPR. A stringent complete response was observed in 58% of patients, 28% achieved VGPR, and 8% attained a partial response (**Table 2**). One patient (1%) was refractory, and 2 patients (3%) died prior to the first evaluation. One of these fatalities was attributed to MAS-*like*, with histopathological evidence found in the liver tissue. Patients who developed MAS-*like* exhibited a significantly lower response compared to controls (≥CR 25% vs. 68%, *p* = 0.008). Furthermore, patients who met all three criteria exhibited a worse response than those who met only two (VGPR or better: 25% vs. 100%, *p* = 0.04).

**Figure 1:**
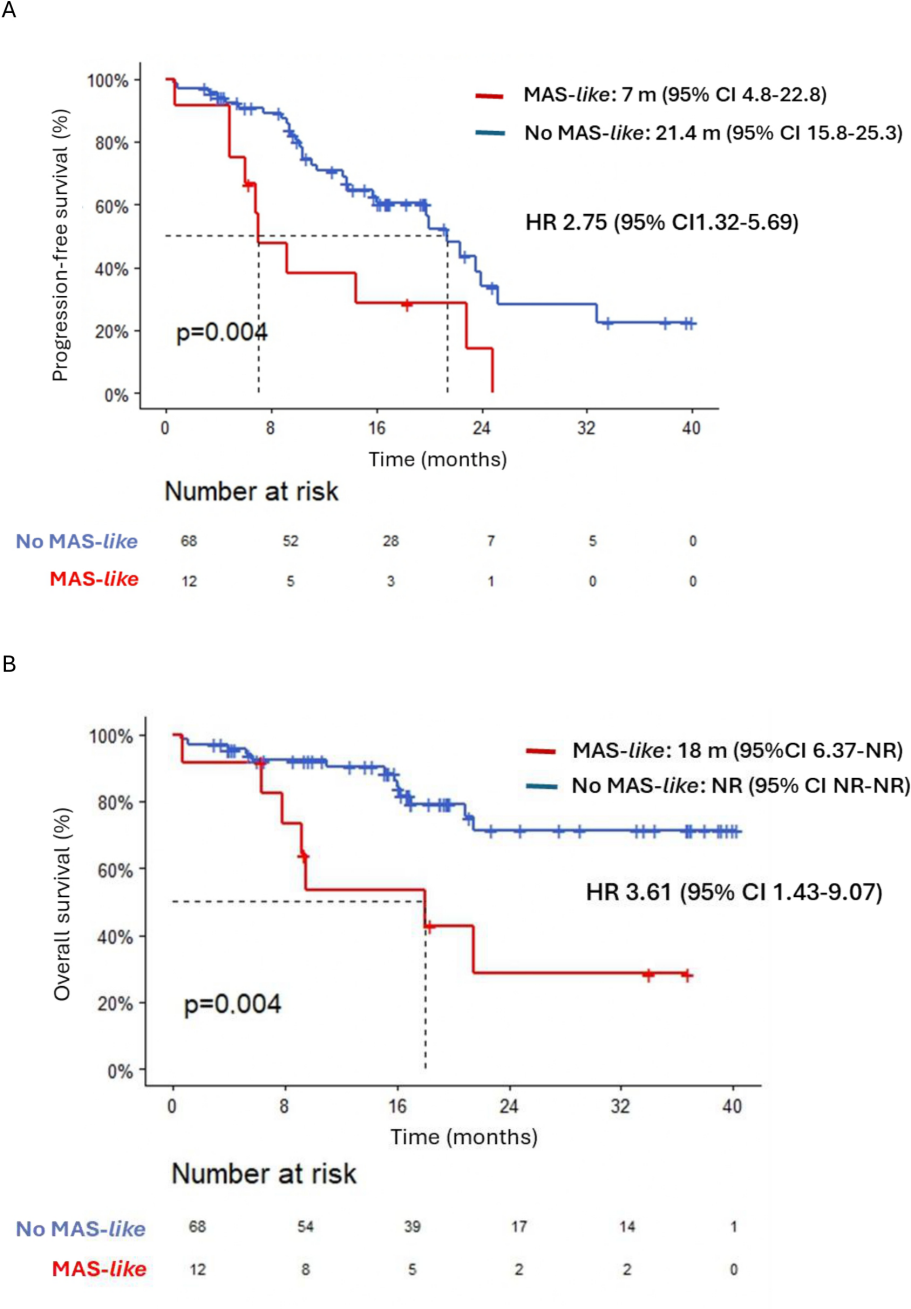
**(A)** Progression-free survival in patients who developed MAS-*like* compared to those who did not. **(B)** Overall survival in patients who developed MAS-*like* compared to those who did not.

**Figure 2:**
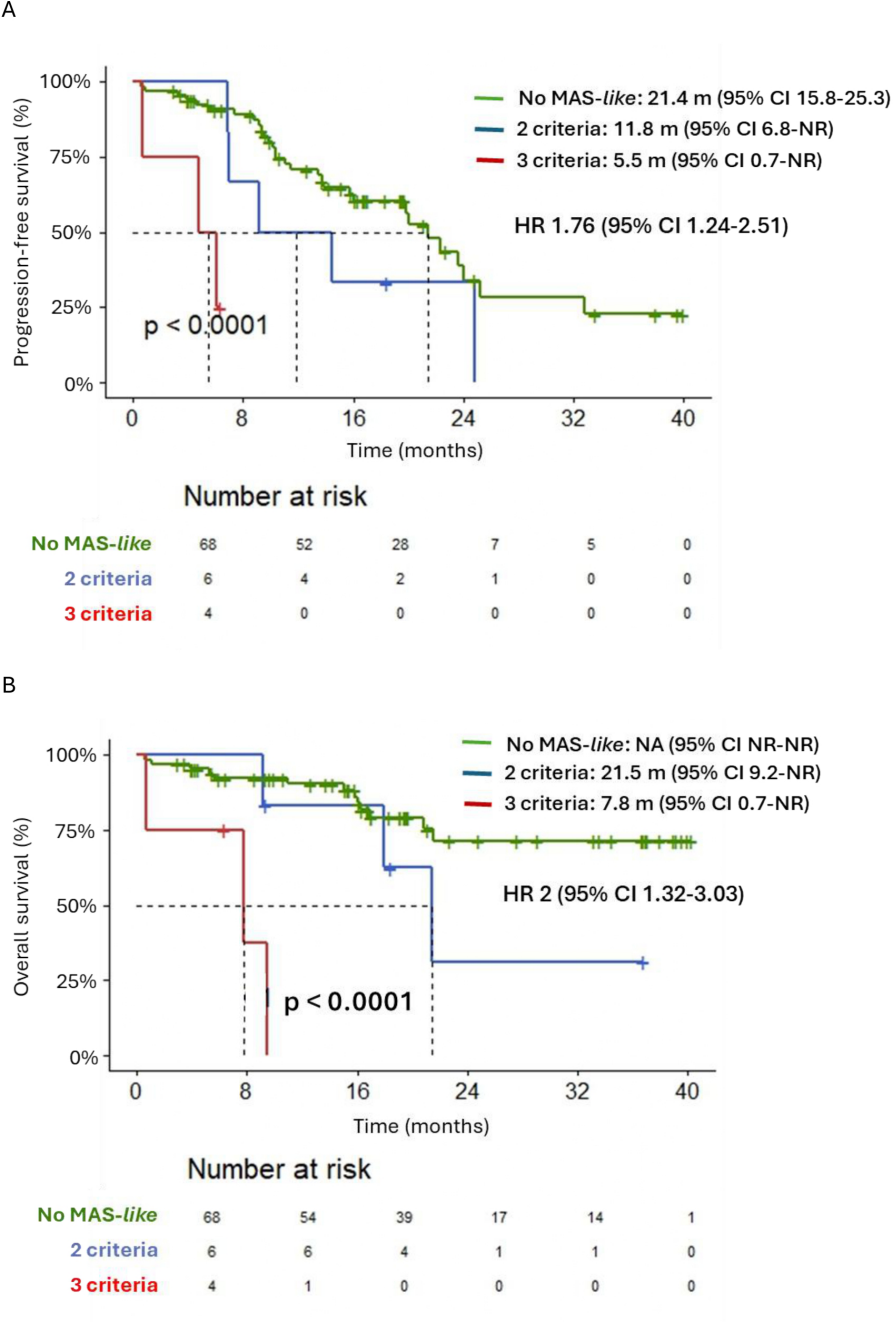
**(A)** Progression-free survival in patients according to the number of University California San Francisco criteria they met (No MAS-*like*, MAS-*like* with 2 criteria and MAS-*like* with 3 criteria). **(B)** Overall survival in patients according to the number of University California San Francisco criteria they met (No MAS-*like*, MAS-*like* with 2 criteria and MAS-*like* with 3 criteria).

Median PFS was shorter in patients who developed MAS-*like* [7 months (95% CI 4.83-22.87) vs. 21.4 months (95% CI 15.77-25.26)], HR 2.75 (95% CI 1.32-5.69); *p* = 0.004 (**Figure 1A**). Median OS for patients who developed MAS-*like* was 18 months (95% CI 6.35-NR), compared to not reached for the control group, HR 3.61 (95% CI 1.43-9.07); *p* = 0.004 (**Figure 1B**). Moreover, patients who met all three UCSF criteria, compared to those who met only two, exhibited significantly poorer PFS [5.45 months (95% CI 0.69-NR) vs 11.83 (95% CI 6.84-NR)]; *p* = 0.012) and OS [7.82 (95% CI 0.69-NR) vs 21.45 (95% CI 9.17-NR)]; *p* = 0.017 (**Figure 2**). Results after FDR correction lose significance due to the small event count. In multivariable analysis, MAS-*like* lost its statistical significance for PFS and OS, unlike other variables such as the ISS, serum monoclonal component, or extramedullary disease (**Supplementary Table 4 and 5**).

## Discussion

Anti-BCMA CAR T-cell therapy has demonstrated a significant impact on the treatment of patients with MM; however, this novel therapeutic approach is associated with a distinct spectrum of immune-mediated adverse events, such as MAS-*like*. Notably, this complication is likely underreported in both clinical trials and real-world studies. In the present cohort of 80 patients treated with the anti-BCMA product ARI0002h, we observed an incidence of 15%, which was correlated with poorer treatment responses and reduced survival outcomes.

The diagnosis of secondary HLH/MAS-*like* remains challenging due to the absence of a definitive diagnostic test and the overlap of its clinical manifestations with other adverse events, such as CRS (21). Consequently, the incidence of MAS-*like* is not consistently reported in clinical trials and varies depending on the diagnostic criteria employed; many reports are based on retrospective analysis. Furthermore, both the type of tumour and the specific CAR T-cell product used are critical factors influencing the development of this complication. Anti-CD22-targeted CAR T-cell therapies have been associated with a notably high incidence of HLH-like reactions, with rates reaching up to 36% in certain cohorts (22, 23). Anti-CD19-based CAR T-cell therapies, in contrast, appear to be associated with a lower incidence of HLH/MAS-like (24). For instance, the reported incidence of HLH in real-world studies of axicabtagene ciloleucel (axi-cel), based on the HLH-2004 criteria, is up to 5% in patients with diffuse large B-cell lymphoma (DLBCL) (25).

MAS-*like* appears to be a relatively common entity observed in patients with MM undergoing CAR-T cell therapy. The incidence rates vary from 4% in the ide-cel clinical trial (1) to approximately 20% in certain real-world cohorts of patients treated with anti-BCMA CAR T-cell products (7, 10). As a potential pathophysiological explanation, the upregulation of major histocompatibility complex (MHC)-II expression on MM cells, triggered by IFN-γ, may contribute to the dysregulated immune response within the pro-inflammatory microenvironment following CAR T-cell infusion (26). The lower incidence of MAS-*like*, according to UCSF criteria, observed in ARI0002h (15%) compared to the 22% reported by Kennedy et al. (7) may be related to the initial fractionated infusion strategy and the withholding of treatment in the presence of adverse events. This hypothesis is supported by the reduced incidence of severe adverse events, such as CRS, in the few products administered in a fractionated schedule, including LCAR-B38M (LEGEND-2 study) and ARI0001 (CART19-BE-01), although differences among constructs should be taken into consideration (17, 27). Differences between MAS-*like* cases reported with other commercial CAR-T products and ARI0002h may be attributed to the distinct diagnostic criteria used. For this study, the UCSF consensus criteria were selected among other available definitions, owing to their clarity and well-defined requirements. Based on our findings, the UCSF criteria appear to be highly sensitive but may prone to overdiagnosing MAS-*like*. Nevertheless, our group posits that early diagnosis—prior to the onset of full organ failure and the severe manifestations of the syndrome—may enhance management and improve patient outcomes (28).

Based on our results, a higher tumour burden, as indicated by an increased serum monoclonal component, and a higher ISS at enrolment, were significantly more frequent in patients who developed MAS-*like*. Indeed, an ISS of III and elevated serum monoclonal component were associated with an increased risk of developing this complication. The involvement of high tumour burden has been previously identified in other hematologic malignancies (29, 30); however, MAS-*like* predisposing factors associated with CAR T-cell therapy have not been well characterized in multiple myeloma. These findings support the hypothesis of an uncontrolled inflammatory response activated by high tumour antigen concentrations (31). The prevalence of extramedullary disease prior to infusion demonstrated a borderline association (p = 0.057), although its potential role in MAS-*like* is supported by previous reports indicating that its presence increases the risk of CRS (32). In this context, extramedullary disease may elicit a MAS-*like* through its tumour microenvironment, which is enriched with M2 polarized tumour-associated macrophages (TAM) that secrete IL-6 (33, 34).

According to the literature, this complication typically manifests in a delayed manner following infusion, most often after the onset of CRS (7, 10, 35). Our findings support this observation, as MAS-*like* syndrome developed later (median of 10 days after the first infusion to meet UCSF criteria, compared to a median of 7 days for CRS), even when the product was administered in progressively increasing doses, as in the case of ARI0002h. The increase in ferritin levels >100 mg/L/h within a 24-hour period was the most sensitive parameter analysed. This finding supports the value of ferritin measurement when CAR-T inflammatory toxicity is suspected, as it may help to identify patients at risk of developing MAS-*like* syndrome. LDH appeared to meet the criteria more frequently than fibrinogen, and a significant poor prognosis was observed in patients who met all three criteria. Patients who met the UCSF criteria exhibited other clinical and laboratory features commonly associated with classical MAS, such as hypertransaminasemia, hypertriglyceridemia, and cytopenias (36, 37). Additionally, most patients showed histopathological findings consistent with MAS.

In line with previous reports, ARI0002h demonstrates favourable efficacy outcomes. The median PFS of the entire cohort was 20 months, with the median OS not reached, which is in line with the outcomes of currently approved CAR-T therapies for MM (1, 9). However, patients who developed MAS-*like* exhibited a poorer response rate (CR 25% vs. 68%) and impaired survival (median PFS 7 months vs. 21.4 months, median OS 18 months vs. not reached). The differences observed were more pronounced in patients who met all three criteria compared to those who met only two (median PFS 5.5 months vs. 11.8 months, median OS 7.8 months vs. 21.5 months). The better outcomes in patients who met only two UCSF criteria may support the necessity of early detection of MAS-*like* syndrome before all manifestations are fully developed. However, subgroup analyses are severely underpowered (12 events, 4 with UCSF = 3). Therefore, results should be interpreted as hypothesis-generating only. Furthermore, the contribution of a higher tumour burden to the poorer outcomes observed in these patients must be taken into account. Together with the limited sample size and the relatively low frequency of MAS-*like* as an adverse event, this may explain the loss of statistical significance in the multivariate analysis.

Our genetic analysis suggests a complex interplay between inherited genetic factors and the development of MAS-like in patients undergoing CAR-T therapy. Variants in UNC13D and PRF1, previously reported to impair perforin activity (20), were not statistically significant predictors on their own. However, when combined with high disease burden and extramedullary disease, they may increase the risk of MAS-like, as suggested by our clinical correlation analysis. These findings reinforce the multifactorial nature of MAS in the setting of advanced therapies such as CAR-T. However, the reduced sample size, together with the limited incidence of MAS-like, hampers the ability to draw definitive conclusions and may increase the influence of outliers.

The treatment of MAS-like syndrome requires the control of the inflammatory loop and close monitoring. There is a wide variety of drugs that are often employed without a standard algorithm. Although controversy exists in some cases, the most frequently used drugs are antibodies or recombinant proteins targeting interleukins, such as anakinra (IL-1) or tocilizumab (IL-6), systemic steroids, ruxolitinib (a JAK inhibitor), emapalumab (an IFN-γ inhibitor), and etoposide (31, 38–42). In this study, most patients who developed MAS-*like* received standard CRS management, which comprises anti-IL6, anti-IL1, and steroids. This is consistent with the initial diagnosis of CRS at the beginning of treatment. Noticeably, a half of patients required two or more drugs (mainly tocilizumab and dexamethasone). The limited use of anakinra may be related to its approval for IEC-HS treatment in our country only after July 2023, by which time most patients had already received ARI0002h. Nonetheless, these observations suggest that CRS and MAS-*like* syndrome share several features of systemic inflammation, in which prompt therapeutic intervention may be crucial to prevent the establishment of a positive feedback loop.

Our study has several limitations. This multicentre study involved a cohort of 80 patients treated with the same anti-BCMA CAR-T therapy, in a context of scarcity of studies focused on this complication. Nevertheless, the retrospective design and the limitation to a single country may introduce potential biases. Moreover, the analytical criteria employed may be insufficient for accurately differentiating between the hyperinflammation associated with CRS and MAS-*like* syndrome. Other criteria with different sensitivity and specificity could be explored prospectively in the future.

In summary, the development of MAS-like is associated with poorer responses and shortened PFS and OS in patients with MM treated with ARI0002h, especially in those who meet all three criteria: elevated ferritin, decreased fibrinogen, and increased LDH. Therefore, further research is required to establish diagnostic criteria that enable early identification and timely therapeutic management of MAS-like.

## Data Availability

The original contributions presented in the study are included in the article/**supplementary material**, further inquiries can be directed to the corresponding author/s.
