# Supplementary figures and images for: Macrophage activation syndrome-like in multiple myeloma patients treated with the academic CAR-T against BCMA ARI0002h
Source: Front Immunol. 2025 Oct 16;16:1654096. doi: 10.3389/fimmu.2025.1654096 (PMC12571847; doi:10.3389/fimmu.2025.1654096)

## Slide 1
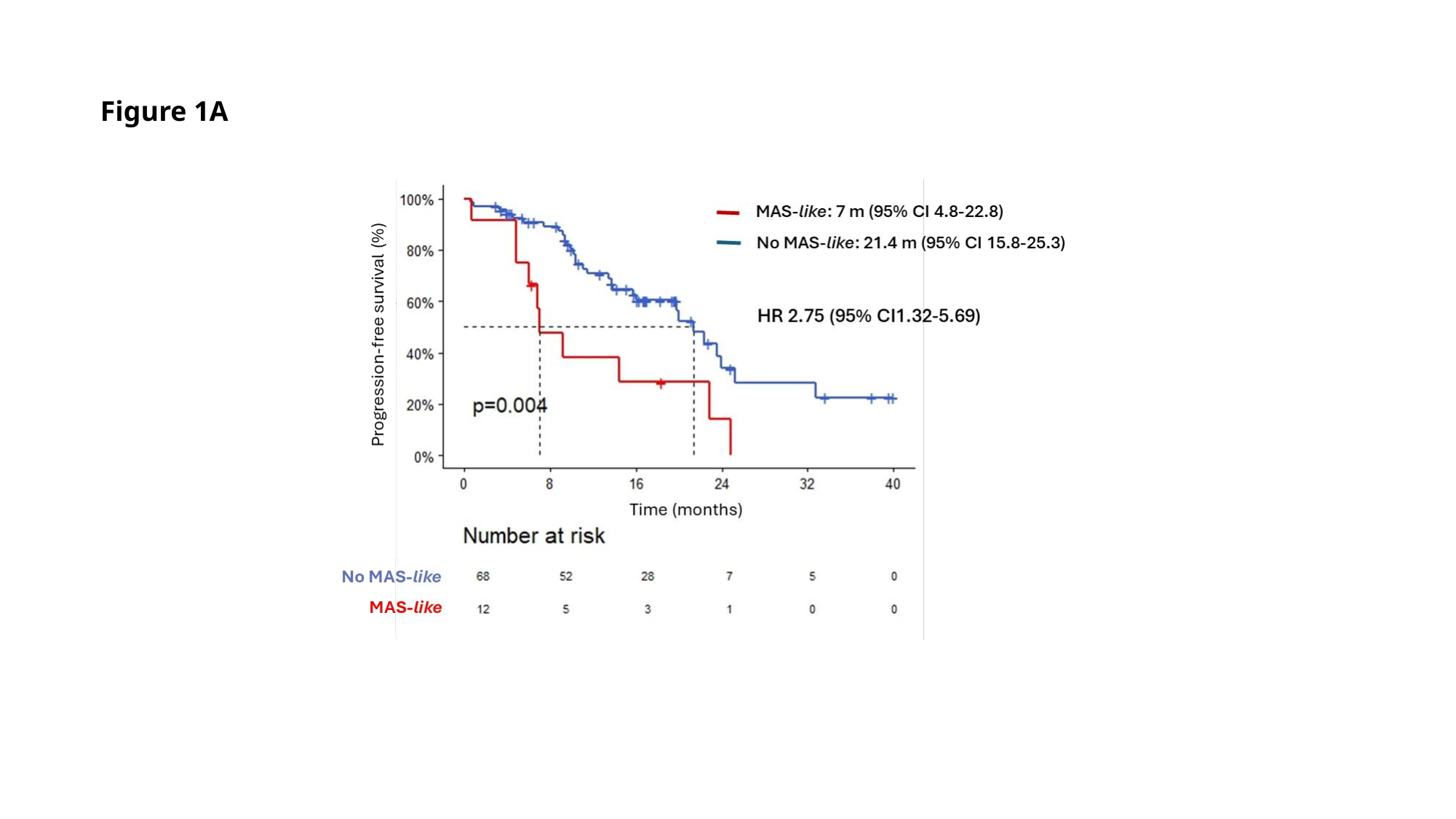

Figure 1A

## Slide 2
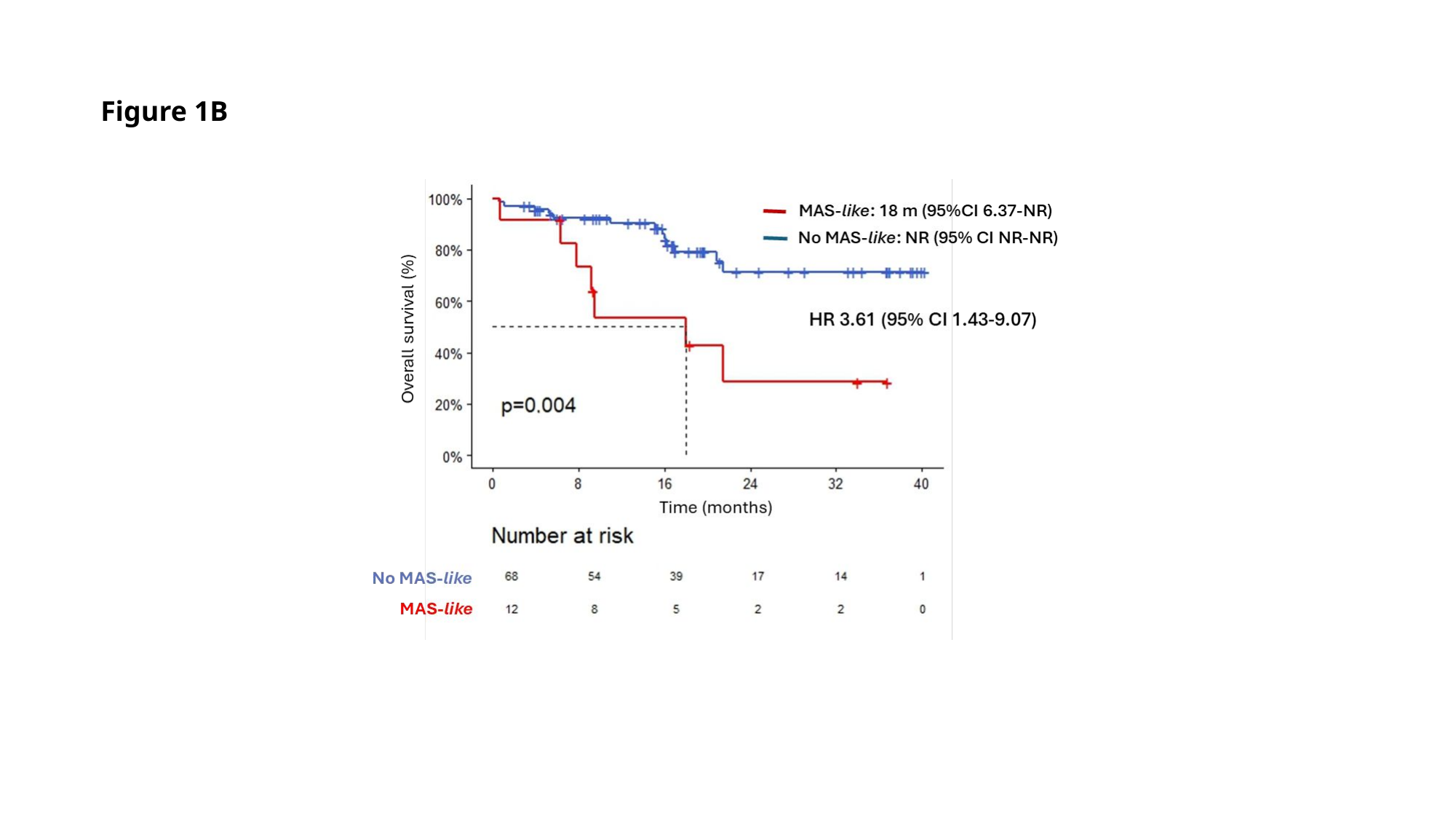

Figure 1B

## Slide 3
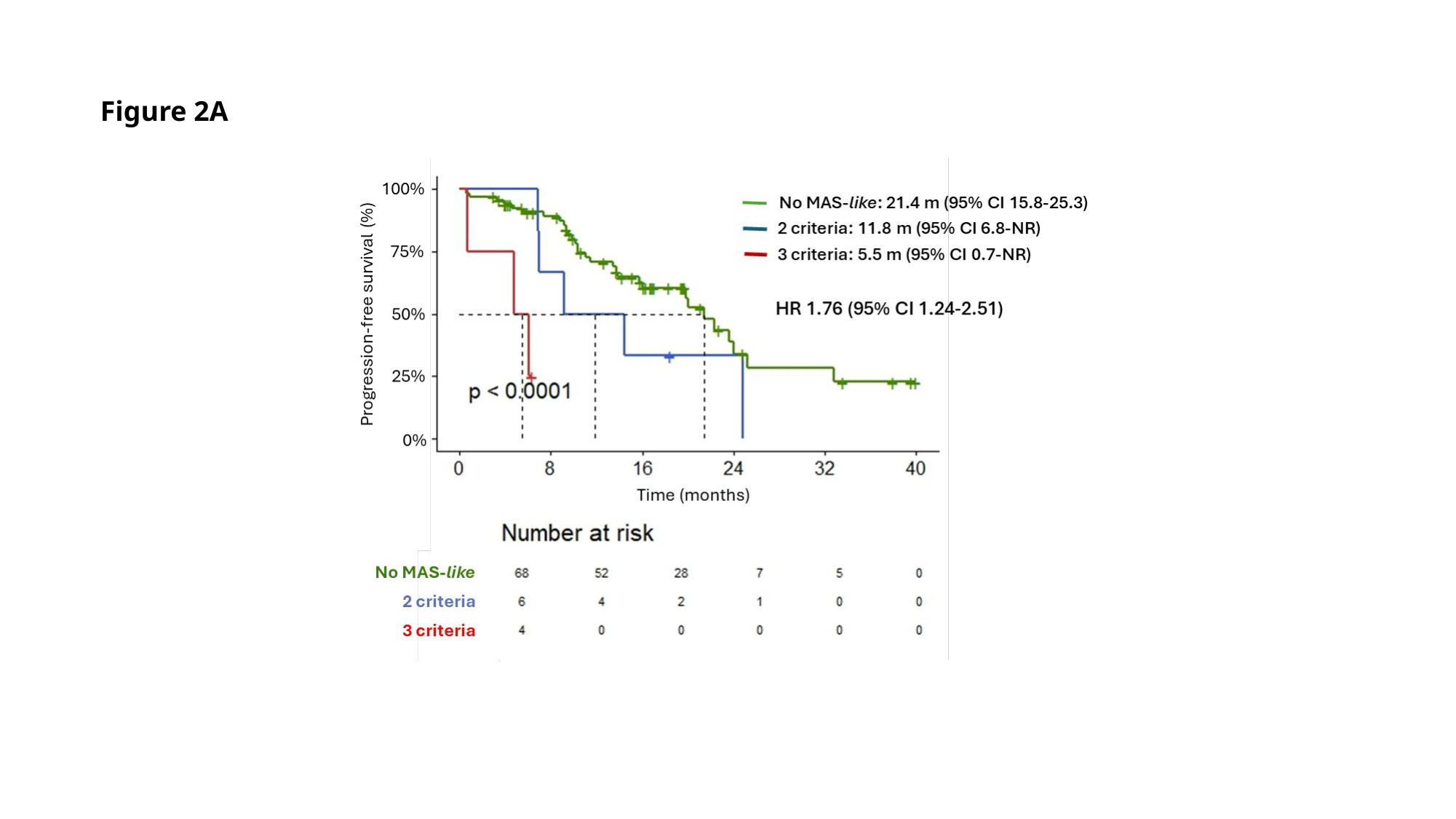

Figure 2A

## Slide 4
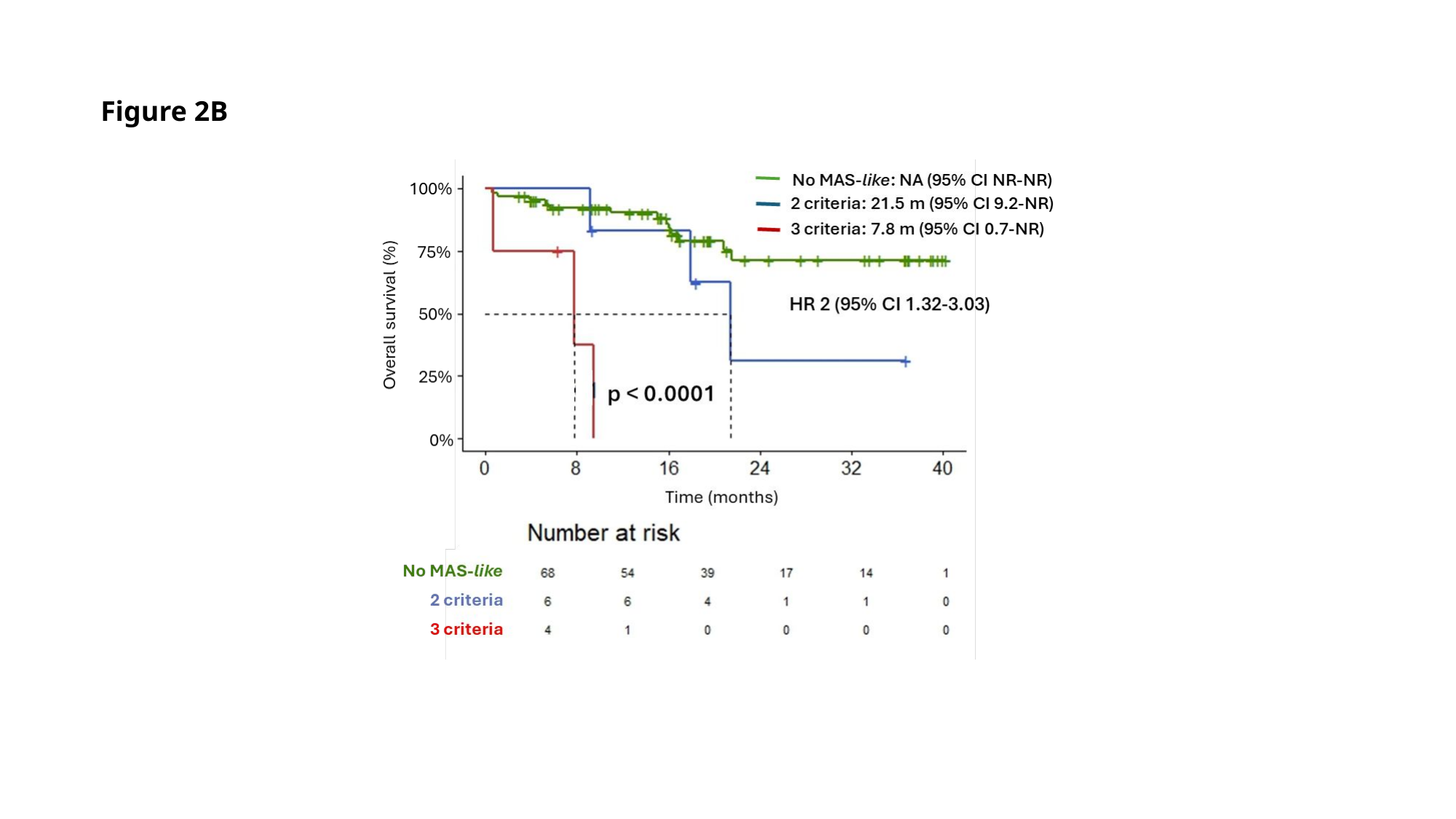

Figure 2B

Supplement: Supplementary file 1 [file Presentation1.pptx]
