# Supplementary material for: Macrophage activation syndrome-like in multiple myeloma patients treated with the academic CAR-T against BCMA ARI0002h
Source: Front Immunol. 2025 Oct 16;16:1654096. doi: 10.3389/fimmu.2025.1654096 (PMC12571847; doi:10.3389/fimmu.2025.1654096)
Supplement: Supplementary file 2 [file Table1.docx]

**SUPPLEMENTARY MATERIAL**

**SUPPLEMENTARY MATERIAL INDEX**

1. **ANNEX**
2. Annex 1
   1. Methods: ARI0002h protocol 3
3. **FIGURES**
4. Figure legends 4
5. Supplementary figures
   1. Figure 1 4
6. **TABLES**
7. Supplementary tables
8. Table 1 5
9. Table 2 6
10. Table 3 9
11. Table 4 10
12. Table 5 12

**Annex 1**

Inclusion and exclusion criteria clinical trial CARTBCMA-HBC-01

Eligible patients for CARTBCMA-HCB-01 had relapsed or refractory multiple myeloma; were aged 18–75 years; had an Eastern Cooperative Oncology Group performance status of 0–2; two or more previous lines of therapy including a proteasome inhibitor, an immunomodulating agent, and an anti-CD38 antibody; refractoriness to the last line of therapy; and measurable disease (serum and urine monoclonal protein >10 g/L or 200 mg/24 h and involved free light chain >100 mg/L) according to the International Myeloma Working Group (IMWG) criteria; and a life expectancy of more than 3 months. Exclusion criteria included previous BCMA-directed therapy and a non-adequate organ system function including an estimated glomerular filtration rate below 50 mL/min.

Reference:

*Oliver-Caldés A, González-Calle V, Cabañas V, Español-Rego M, Rodríguez-Otero P, Reguera JL, et al. Fractionated initial infusion and booster dose of ARI0002h, a humanised, BCMA-directed CAR T-cell therapy, for patients with relapsed or refractory multiple myeloma (CARTBCMA-HCB-01): a single-arm, multicentre, academic pilot study. Lancet Oncol. August 2023;24(8):913-24.*

**SUPPLEMENTARY FIGURES**

**FIGURE LEGENDS**

**Figure 1.** Comparison between ferritin levels prior to the onset of MAS-like and its maximum (A). Pearson correlation analysis between the maximum ferritin level and the minimum fibrinogen level reached (B).

**FIGURES**

Figure 1

A


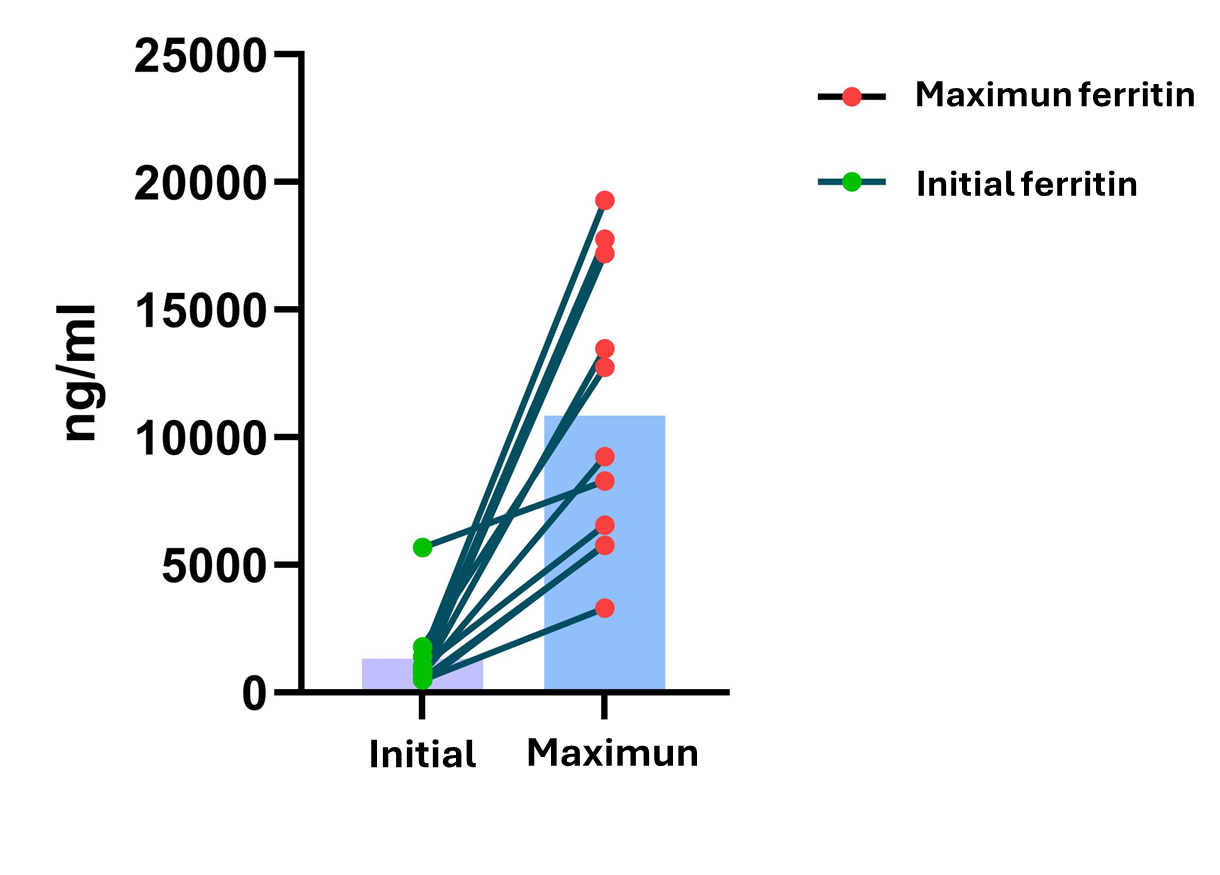


B


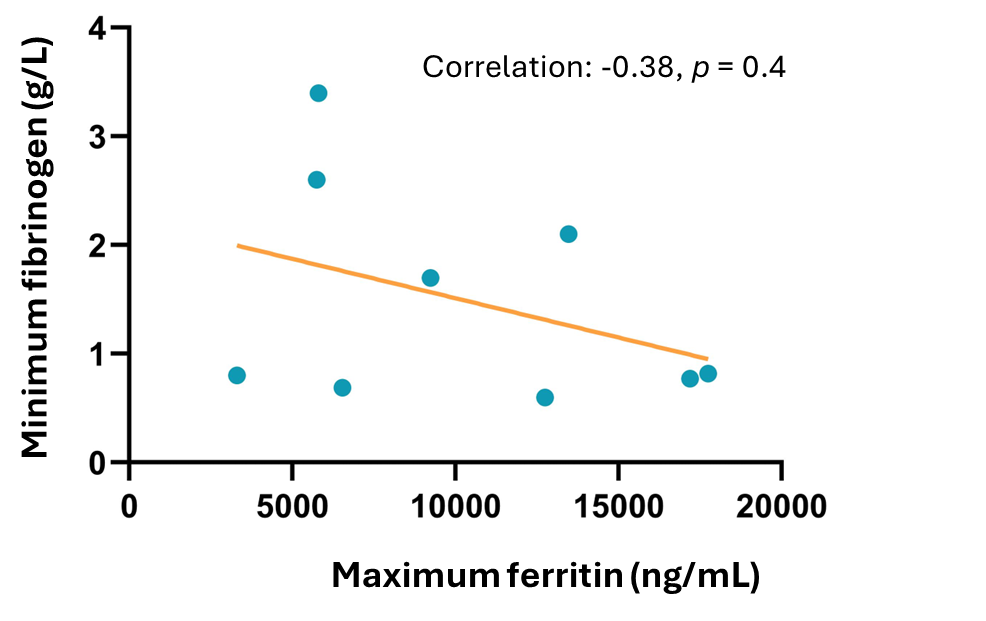


**SUPPLEMENTARY TABLES**

**Table 1.** Hemophagocytic lymphohistiocytosis related gene panel

| **AP3B1** | **GATA2** | **LYST** | **PRF1** | **STK4** |
| --- | --- | --- | --- | --- |
| **AP3D1** | **HAVCR2** | **MAGT1** | **PRKCD** | **STX11** |
| **CD70** | **IKZF2** | **MCM10** | **PTEN** | **STXBP2** |
| **CDC42** | **IKZF3** | **MCM4** | **RAB27a** | **TET2** |
| **CORO1A** | **IL2RB** | **NFKB1** | **RASGRP1** | **TNFRSF7** |
| **CTLA4** | **IRF8** | **NLRC4** | **RHOG** | **TNFRSF9** |
| **CTPS1** | **ITK** | **PIK3CD** | **RLTPR** | **UNC13D** |
| **FAAP24** | **LIG4** | **PIK3CG** | **SH2D1A** | **XIAP** |
| **FCGR3A** | **LRBA** | **PIK3R1** | **SLC7A7** | **ZAP70** |

**Table 2.** Variants identified in patients with MAS-*like* (A) and patients without MAS-*like* (B).

A.

| Patient | Gene | Coding DNA | Protein | Zygosity | Variant type | Transcript accession number | Total frequency gnomAD v4.1.0 (%) | Prediction SIFT/Polyphen | ClinVar (number of submissions) | Franklin |
| --- | --- | --- | --- | --- | --- | --- | --- | --- | --- | --- |
| 1 | RASGRP1 | c.1571C>G | p.(Ala524Gly) | Het | Missense | NM_005739.4 | 0.001178 | deleterious_low_confidence / benign | Absent | VUS |
| 2 | UNC13D | c.2219C>T | p.(Thr740Met) | Het | Missense | NM_199242.3 | 0.005301 | Deleterious / probably_damaging | VUS(3) | VUS |
| 3 | PRF1 | c.272C>T | p.(Ala91Val) | Het | Missense | NM_001083116.3 | 3.540 | Deleterious / possibly_damaging | VUS(4)/ LB(3)/B(3) | VUS |
| 4 | AP3D1 | c.1605G>T | p.(Gln535His) | Het | Missense | NM_001261826.3 | Absent | deleterious_low_confidence / possibly_damaging | Absent | VUS |
| 5 | PRF1 | c.272C>T | p.(Ala91Val) | Het | Missense | NM_001083116.3 | 3.540 | Deleterious / possibly_damaging | VUS(4)/ LB(3)/B(3) | VUS |
| 6 | LYST | c.143A>G | p.(His48Arg) | Het | Missense | NM_000081.4 | 0.02069 | tolerated_low_confidence / benign | VUS(5) | LB |
|  | UNC13D | c.610A>G | p.(Met204Val) | Het | Missense | NM_199242.3 | 0.1110 | Deleterious / benign | VUS(6)/LB(1) | B |
| 7 | - | - | - | - | - | - | - | - | - | - |
| 8 | - | - | - | - | - | - | - | - | - | - |
| 9 | - | - | - | - | - | - | - | - | - | - |
| 10 | RASGRP1 | c.234C>A | p.(Asn78Lys) | Het | Missense | NM_005739.4 | 0.0002480 | tolerated_low_confidence / benign | Absent | VUS |
| 11 | - | - | - | - | - | - | - | - | - | - |
| 12 | - | - | - | - | - | - | - | - | - | - |

B.

| Patient | Gene | Coding DNA | Protein | Zygosity | Variant type | Transcript accession number | Total frequency gnomAD v4.1.0 (%) | Prediction SIFT/Polyphen | ClinVar (number of submissions) | Franklin |
| --- | --- | --- | --- | --- | --- | --- | --- | --- | --- | --- |
| 13 | - | - | - | - | - | - | - | - | - | - |
| 14 | PIK3CD | c.58G>A | p.(Val20Ile) | Het | Missense | NM_005026.5 | 0.01059 | tolerated_low_confidence / benign | VUS(1) | VUS |
|  | GATA2 | c.374C>T | p.(Pro125Leu) | Het | Missense | NM_032638.5 | Absent | Tolerated / benign | Absent | VUS |
| 15 | IL2RB | c.508C>T | p.(Arg170Trp) | Het | Missense | NM_000878.5 | 0.002044 | Tolerated / probably_damaging | VUS(1) | VUS |
| 16 | - | - | - | - | - | - | - | - | - | - |
| 17 | - | - | - | - | - | - | - | - | - | - |
| 18 | PRF1 | c.272C>T | p.(Ala91Val) | Het | Missense | NM_001083116.3 | 3.540 | Deleterious / possibly_damaging | VUS(4)/ LB(3)/B(3) | VUS |
| 19 | LRBA | c.1399A>G | p.(Met467Val) | Het | Missense | NM_001364905.1 | 0.3285 | deleterious_low_confidence / benign | VUS(7)/ LB(2)/B(1) | B |
| 20 | - | - | - | - | - | - | - | - | - | - |
| 21 | CD70 | c.437G>T | p.(Ser146Ile) | Het | Missense | NM_001252.5 | 0.04770 | Deleterious / benign | Absent | VUS |
| 22 | TNFRSF9 | c.559A>G | p.(Ile187Val) | Het | Missense | NM_001561.6 | Absent | Tolerated / benign | Absent | VUS |
| 23 | - | - | - | - | - | - | - | - | - | - |
| 24 | LIG4 | c.2465C>T | p.(Ser822Leu) | Het | Missense | NM_206937.2 | 0.01159 | Tolerated / - | VUS(3) | VUS |
|  | ZAP70 | c.664T>C | p.(Cys222Arg) | Het | Missense | NM_001079.4 | 0.00006195 | Deleterious / benign | Absent | VUS |
| 25 | - | - | - | - | - | - | - | - | - | - |
| 26 | PRF1 | c.272C>T | p.(Ala91Val) | Het | Missense | NM_001083116.3 | 3.540 | Deleterious / possibly_damaging | VUS(4)/ LB(3)/B(3) | VUS |
| 27 | - | - | - | - | - | - | - | - | - | - |
| 28 | PRF1 | c.272C>T | p.(Ala91Val) | Het | Missense | NM_001083116.3 | 3.540 | Deleterious / possibly_damaging | VUS(4)/ LB(3)/B(3) | VUS |
|  | UNC13D | c.869C>T | p.(Ser290Leu) | Het | Missense | NM_199242.3 | 0.007875 | Deleterious / benign | VUS(4) | VUS |
| 29 | PRF1 | c.82C>T | p.(Arg28Cys) | Het | Missense | NM_001083116.3 | 0.08304 | Deleterious / probably_damaging | VUS(6)/B(1) | B |
| 30 | AP3D1 | c.1985A>G | p.(Glu662Gly) | Het | Missense | NM_001261826.3 | 0.0008137 | deleterious_low_confidence / possibly_damaging | Absent | VUS |
| 31 | FAAP24 | c.613C>T | p.(Gln205Ter) | Het | Nonsense | NM_152266.5 | 0.0001239 | -/- | Absent | VUS |
|  | LRBA | c.5030A>G | p.(Asn1677Ser) | Het | Missense | NM_001364905.1 | 0.08729 | deleterious_low_confidence / probably_damaging | B(4), VUS(3) | B |
|  | PIK3CG | c.896A>T | p.(Asn299Ile) | Het | Missense | NM_001282426.2 | Absent | Deleterious / possibly_damaging | Absent | VUS |
| 32 | PRF1 | c.272C>T | p.(Ala91Val) | Het | Missense | NM_001083116.3 | 3.540 | Deleterious / possibly_damaging | VUS(4)/ LB(3)/B(3) | VUS |
| 33 | MCM4 | c.658A>G | p.(Lys220Glu) | Het | Missense | NM_182746.3 | 0.005601 | Tolerated / benign | VUS(1) | VUS |
| 34 | - | - | - | - | - | - | - | - | - | - |
| 35 | AP3D1 | c.1183G>A | p.(Glu395Lys) | Het | Missense | NM_001261826.3 | 0.0005575 | deleterious_low_confidence / probably_damaging | Absent | VUS |
|  | LRBA | c.7840A>G | p.(Thr2614Ala) | Het | Missense | NM_001364905.1 | 0.008630 | deleterious_low_confidence / benign | VUS(3) | VUS |
| 36 | - | - | - | - | - | - | - | - | - | - |
| 37 | LYST | c.6460G>A | p.(Asp2154Asn) | Het | Missense | NM_000081.4 | 0.01078 | deleterious_low_confidence / benign | VUS(6) | VUS |
|  | HAVCR2 | c.461C>G | p.(Thr154Ser) | Het | Missense | NM_032782.5 | 0.1729 | Tolerated / possibly_damaging | Absent | VUS |
| 38 | UNC13D | c.2173G>C | p.(Glu725Gln) | Het | Missense | NM_199242.3 | 0.0007673 | Tolerated / probably_damaging | Absent | VUS |
| 39 | - | - | - | - | - | - | - | - | - | - |
| 40 | TNFRSF9 | c.559A>G | p.(Ile187Val) | Het | Missense | NM_001561.6 | Absent | Tolerated / benign | Absent | VUS |
| 41 | - | - | - | - | - | - | - | - | - | - |
| 42 | LYST | c.4705A>C | p.Asn1569His | Het | Missense | NM_000081.4 | 0.008551 | Deleterious_low_confidence / probably_damaging | VUS(7)/LB(1) | VUS |
| 43 | ITK | c.808G>T | p.(Ala270Ser) | Het | Missense | NM_005546.4 | 0.00006196 | Tolerated / benign | Absent | VUS |
| 44 | - | - | - | - | - | - | - | - | - | - |

CRS: cytokine release syndrome, Het: Heterozygous, VUS: Variant of uncertain significance, B: Benign

**Table 3.** Univariate (A) and multivariate (B) logistic regression analysis to predict MAS-like syndrome.

A.

| **Variables** | **Univariate analysis** | | | |
| --- | --- | --- | --- | --- |
|  | **OR** | **Inferior 95% CI** | **Superior 95% CI** | ***p* value** |
| **Age** >**65** | 1.600 | 0.455 | 5.620 | 0.666 |
| **Female** | 0.597 | 0.164 | 2.170 | 0.433 |
| **Prior lines ≥3** | 1.190 | 0.874 | 1.630 | 0.266 |
| **Triple refractory** | 1.840 | 0.439 | 7.730 | 0.404 |
| **ISS** **III at inclusion**^1^ | 6.720 | 1.720 | 26.30 | **0.006** |
| **No IgG heavy chain** | 0.296 | 0.074 | 1.190 | 0.086 |
| **Lambda light chain** | 0.853 | 0.246 | 2.950 | 0.801 |
| **Plasma cells in bone marrow ≥10%** | 1.100 | 0.315 | 3.830 | 0.882 |
| **Monoclonal serum component >30 g/l** | 14.60 | 3.280 | 65.40 | **<0.001** |
| **Monoclonal component, urine** | 1 | 0.985 | 1.020 | 0.910 |
| **Involved light chain serum >100 mg/l** | 1.410 | 0.148 | 13.40 | 0.764 |
| **High risk cytogenetics**^2^ | 0.578 | 0.139 | 2.400 | 0.451 |
| **Extramedullary disease** | 3.700 | 0.992 | 13.80 | 0.051 |
| **HSCT**^3^ | 0.331 | 0.073 | 1.500 | 0.152 |
| **Bridging treatment**  B. | 1.170 | 0.338 | 4.070 | 0.801 |
| **Variables** | **Multivariate análisis** | | | |
|  | **OR** | **Inferior 95% CI** | **Superior 95% CI** | ***p* value** |
| **ISS III at inclusion**^1^ | 2.810 | 0.472 | 16.70 | 0.257 |
| **Monoclonal serum component >30 g/l** | 10.10 | 1.670 | 61.50 | **0.012** |

^1^ISS: International Staging System. ^2^High risk cytogenetics: defined by the presence of del(17p), gain(1q) or t(4;14).^3^HSCT: hematopoietic stem cell transplantation.

**Table 4.** Univariate (A) and multivariate (B) Cox regression analysis of PFS

A.

| **Variables** | **Univariate analysis** | | | |
| --- | --- | --- | --- | --- |
|  | **HR** | **Inferior 95% CI** | **Superior 95% CI** | ***p* value** |
| **Age** >**65** | 0.624 | 0.313 | 1.245 | 0.181 |
| **Female** | 1.102 | 0.582 | 2.083 | 0.766 |
| **Prior lines ≥3** | 1.190 | 0.874 | 1.630 | 0.266 |
| **Triple refractory** | 1.357 | 0.704 | 2.615 | 0.361 |
| **ISS** **III at inclusion**^1^ | 2.491 | 1.263 | 4.911 | **0.008** |
| **No IgG heavy chain** | 0.908 | 0.485 | 1.698 | 0.763 |
| **Lambda light chain** | 0.999 | 0.999 | 1 | 0.304 |
| **Plasma cells in bone marrow ≥10%** | 1.409 | 0.750 | 2.646 | 0.286 |
| **Monoclonal serum component >30 g/l** | 2.947 | 1.415 | 6.137 | **0.004** |
| **Monoclonal component, urine** | 1 | 0.985 | 1.020 | 0.910 |
| **Involved light chain serum >100 mg/l** | 1.276 | 0.382 | 4.260 | 0.691 |
| **High risk cytogenetics**^2^ | 2.038 | 1.074 | 3.865 | **0.029** |
| **Extramedullary disease** | 2.216 | 1.112 | 4.416 | **0.023** |
| **HSCT**^3^ | 1.085 | 0.537 | 2.191 | 0.821 |
| **Bridging treatment** | 2.229 | 1.167 | 4.256 | **0.015** |
| **MAS-like**^4^ | 2.75 | 1.328 | 5.694 | **0.006** |
| B. |  |  |  |  |
| **Variables** | **Multivariate analysis** | | | |
|  | **OR** | **Inferior 95% CI** | **Superior 95% CI** | ***p* value** |

| ISS III at inclusion^1^ | 1.208 | 0.487 | 2.996 | 0.683 |
| --- | --- | --- | --- | --- |
| **Monoclonal serum component >30 g/l** | 1.021 | 1.002 | 1.040 | **0.030** |
| **High risk cytogenetics**^2^ | 1.916 | 0.927 | 3.958 | 0.079 |
| **Extrameullary disease** | 1.837 | 0.851 | 3.962 | 0.121 |
| **Bridging treatment** | 2.317 | 0.993 | 5.404 | 0.052 |
| **MAS-*like***^4^ | 1.145 | 0.353 | 3.708 | 0.821 |

^1^ISS: International Staging System. ^2^High risk cytogenetics: defined by the presence of del(17p), gain(1q) or t(4;14). ^3^HSCT: hematopoietic stem cell transplantation. ^4^MAS-*like*: Macrophage activation syndrome.

**Table 5.** Univariate (A) and multivariate (B) Cox regression analysis of OS.

A.

| **Variables** | **Univariate analysis** | | | |
| --- | --- | --- | --- | --- |
|  | **HR** | **Inferior 95% CI** | **Superior 95% CI** | ***p* value** |
| **Age** >**65** | 0.618 | 0.224 | 1.704 | 0.352 |
| **Female** | 1.936 | 0.801 | 4.679 | 0.142 |
| **Prior lines ≥3** | 1.108 | 0.892 | 1.377 | 0.355 |
| **Triple refractory** | 1.962 | 0.741 | 5.194 | 0.174 |
| **ISS** **III at inclusion**^1^ | 4.161 | 1.603 | 10.80 | **0.003** |
| **No IgG heavy chain** | 0.751 | 0.310 | 1.815 | 0.523 |
| **Lambda light chain** | 1.236 | 0.514 | 2.972 | 0.636 |
| **Plasma cells in bone marrow ≥10%** | 1.310 | 0.532 | 3.228 | 0.556 |
| **Monoclonal serum component >30 g/l** | 2.947 | 1.415 | 6.137 | **0.004** |
| **Monoclonal component, urine** | 1.003 | 0.991 | 1.015 | 0.655 |
| **Involved light chain serum >100 mg/l** | 0.692 | 0.151 | 3.172 | 0.635 |
| **High risk cytogenetics**^2^ | 2.845 | 1.170 | 6.918 | **0.021** |
| **Extramedullary disease** | 2.716 | 1.080 | 6.826 | **0.033** |
| **HSCT**^3^ | 0.697 | 0.246 | 1.974 | 0.496 |
| **Bridging treatment** | 1.317 | 0.543 | 3.193 | 0.542 |
| **MAS-like**^4^ | 3.608 | 1.434 | 9.079 | **0.006** |
| B. |  |  |  |  |
| **Variables** | **Multivariate analysis** | | | |
|  | **OR** | **Inferior 95% CI** | **Superior 95% CI** | ***p* value** |

| ISS III at inclusion | 3.160 | 1.050 | 9.515 | 0.040 |
| --- | --- | --- | --- | --- |
| **Monoclonal serum component >30 g/l** | 0.974 | 0.226 | 4.198 | 0.972 |
| **High risk cytogenetics** | 3.975 | 1.376 | 11.49 | **0.010** |
| **Extrameullary disease** | 3.583 | 1.286 | 9.987 | **0.014** |
| **MAS-*like*** | 2.695 | 0.640 | 11.34 | 0.176 |

^1^ISS: International Staging System. ^2^High risk cytogenetics: defined by the presence of del(17p), gain(1q) or t(4;14). ^3^HSCT: hematopoietic stem cell transplantation. ^3^MAS-*like*: Macrophage activation syndrome.
